# Robotic Versus Thoracoscopic Sub-lobar Resection for Octogenarians with Clinical Stage IA Non-small Cell Lung Cancer: A Propensity Score-Matched Real-World Study

**DOI:** 10.1245/s10434-023-14689-5

**Published:** 2023-12-10

**Authors:** Hanbo Pan, Ningyuan Zou, Yu Tian, Yaofeng Shen, Hang Chen, Hongda Zhu, Jiaqi Zhang, Weiqiu Jin, Zenan Gu, Junwei Ning, Long Jiang, Jia Huang, Qingquan Luo

**Affiliations:** 1 Shanghai Lung Cancer Center, Shanghai Chest Hospital, Shanghai Jiao Tong University School of Medicine, Shanghai, China; 2 Department of Anesthesiology, Shanghai Chest Hospital, Shanghai Jiao Tong University, Shanghai, China; 3 Department of Cardiothoracic Surgery, The Affiliated Lihuili Hospital of Ningbo University, Zhejiang, China; 4 Department of Thoracic Surgery, Shanghai Tongren Hospital, Shanghai Jiao Tong University School of Medicine, Shanghai, China

**Keywords:** Non-small cell lung cancer, Octogenarians, Robotic-assisted thoracoscopic surgery, Video-assisted thoracoscopic surgery, Sub-lobar resection, Lng-term outcomes

## Abstract

**Background:**

Minimally invasive sub-lobectomy is sufficient in treating small early-stage non-small cell lung cancer (NSCLC). However, comparison of the feasibility and oncologic efficacy between robot-assisted thoracoscopic surgery (RATS) and video-assisted thoracoscopic surgery (VATS) in performing sub-lobectomy for early-stage NSCLC patients age 80 years or older is scarce.

**Methods:**

Octogenarians with clinical stage IA NSCLC (tumor size, ≤ 2 cm) undergoing minimally invasive wedge resection or segmentectomy at Shanghai Chest Hospital from 2011 to 2020 were retrospectively reviewed from a prospectively maintained database. Propensity score-matching (PSM) with a RATS versus VATS ratio of 1:4 was performed. Perioperative and long-term outcomes were analyzed.

**Results:**

The study identified 594 patients (48 RATS and 546 VATS patients), and PSM resulted in 45 cases in the RATS group and 180 cases in the VATS group. The RATS patients experienced less intraoperative bleeding (60 mL [interquartile range (IQR), 50–100 mL] vs. 80 mL [IQR, 50–100 mL]; *P* = 0.027) and a shorter postoperative hospital stay (4 days [IQR, 3–5 days] vs. 5 days [IQR, 4–6 days]; *P* = 0.041) than the VATS patients. The two surgical approaches were comparable concerning other perioperative outcomes and postoperative complications (20.00% vs. 26.11%; *P* = 0.396). Additionally, during a median follow-up period of 66 months, RATS and VATS achieved comparable 5-year overall survival (90.48% vs. 87.93%; *P* = 0.891), recurrence-free survival (83.37% vs. 83.18%; *P* = 0.782), and cumulative incidence of death. Further subgroup comparison also demonstrated comparable long-term outcomes between the two approaches. Finally, multivariate Cox analysis indicated that the surgical approach was not independently correlated with long-term outcomes.

**Conclusions:**

The RATS approach shortened the postoperative hospital stay, reduced intraoperative bleeding by a statistically notable but clinically insignificant amount, and achieved long-term outcomes comparable with VATS in performing sub-lobectomy for octogenarians with early-stage small NSCLC.

**Supplementary Information:**

The online version contains supplementary material available at 10.1245/s10434-023-14689-5.

Currently, non-small cell lung cancer (NSCLC) remains one of the leading causes of cancer mortality worldwide.^1,2^ With the increasing prevalence of thin-section thoracic computed tomography (CT) screening and improvements in diagnostic methods, the early diagnosis rate for early-stage small NSCLC has risen sharply.^3^

During the last decade, the practical indications of sub-lobar resections were extended to early-stage NSCLC and are approved for patients if a small peripheral tumor without lymph node (LN) involvement is found.^4^ Recently, the promising results indicated by the JCOG0802 and CALGB140503 studies have confirmed that sub-lobar resection is not inferior to lobectomy, with improved 5-year overall survival (OS), and thus can be considered one of the standard treatments for peripheral stage IA NSCLC (tumor size, ≤ 2 cm).^5,6^

With the longer life span, the prevalence of NSCLC has increased sharply among octogenarians. This demographic group often presents with multiple comorbidities and reduced cardiopulmonary reserve, rendering them susceptible to a decline in respiratory function.^7,8^ Consequently, these patients are assumed to be at higher risk for lobectomy, with approximately two- to fourfold greater perioperative mortality than patients younger than 80 years.^9–12^

Notably, prior research has directly compared the long-term prognosis between octogenarians with NSCLC who underwent surgical resection and individuals younger than 80 years, consistently showing that the elderly cohort experienced significantly poorer long-term outcomes.^10,13^ It is believed that octogenarians may derive more significant benefits from limited lung parenchyma resection than younger individuals, and both the JCOG0802 and CALGB140503 trials have shown that sub-lobar resection mitigates respiratory function loss compared with lobectomy.^5,6,14,15^

Notably, previous publications have consistently reported significant improvements in perioperative outcomes and potential long-term survival advantages associated with sub-lobar resection over lobar resection for very old patients with early-stage NSCLC, highlighting the effectiveness of lung-sparing surgery in such cases.^8,10,14,16^ Given this, there is a pressing need to determine the most suitable surgical approach for performing sub-lobar resection for octogenarians compared with individuals younger than 80 years.^8,10,14^

Currently, the open thoracotomy approach has been gradually replaced by minimally invasive surgery (MIS) techniques for segmentectomy and wedge resection in treating early-stage NSCLC.^13,17^ Previous publications have shown that video-assisted thoracoscopic surgery (VATS) sub-lobar resection has many superiorities over traditional thoracotomy, including fewer postoperative complications, faster recoveries, less surgery-related pain, and better qualities of life.^13,18,19^

In recent years, robot-assisted thoracoscopic surgery (RATS), an innovative MIS approach first introduced to the thoracic surgical field in 2002, has become increasingly prevalent.^3,7,20^ The robot-assisted surgical system possesses a high-quality, three-dimensional (3D), 10-fold magnified surgical view as well as highly flexible, maneuverable, and stable robot arms, providing operators with great convenience in performing surgical resection and thus notably reducing unnecessary injury.^3,21^ Given these characteristics, RATS may be a more suitable approach than VATS for very old patients who frequently experience postoperative complications, slow recoveries, and poor outcomes.^7^

Previous studies have indicated that RATS could reduce the surgical duration, conversion rate, postoperative complications, and postoperative hospital stay and achieve long-term outcomes comparable with VATS in performing lobectomy for very old NSCLC patients.^7,22,23^ However, the feasibility and oncologic efficacy of RATS for sub-lobar resection in octogenarians with early-stage small NSCLC have never been assessed, and whether RATS exhibits a benefit over VATS for these patients remains unrevealed.

This study retrospectively compared RATS with VATS sub-lobar resection for octogenarians with early-stage small NSCLC, aiming to assess the feasibility and oncologic efficacy of RATS for these patients.

## Methods

### Study Design and Patients

**Fig. 1:**
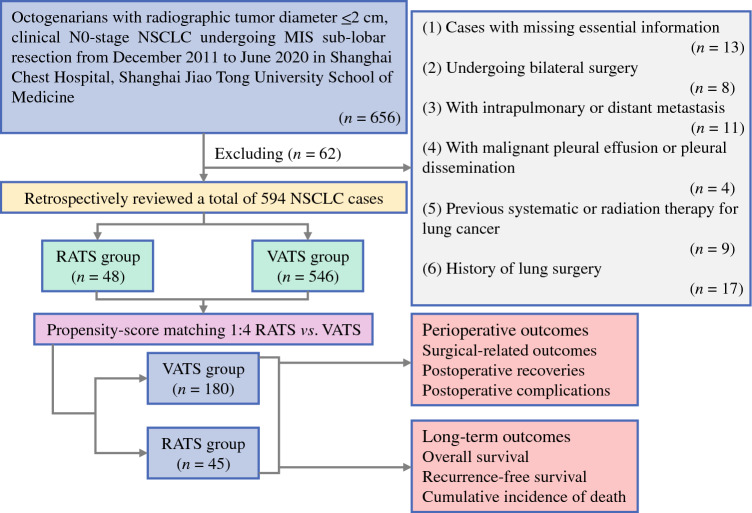
Flow chart of patient selection. NSCLC, non-small cell lung cancer; MIS, minimally invasive surgery; RATS, robot-assisted thoracoscopic surgery; VATS, video-assisted thoracoscopic surgery

The current study was approved by the Institutional Review Board of Shanghai Chest Hospital (approval no. IS23039). In this study, all procedures involving human participants followed the Declaration of Helsinki. Patients 80 years old or older who received MIS sub-lobar resection from December 2011 to June 2020 at Shanghai Chest Hospital were retrospectively reviewed from a prospectively maintained database (Fig. 1). The exclusion criteria ruled out cases with missing essential information, patients undergoing bilateral surgery, patients with intrapulmonary or distant metastasis, patients with malignant pleural effusion or pleural dissemination, patients with previous systematic or radiation therapy for lung cancer, and patients with a history of lung surgery.

The selection of the sub-lobar resection approaches was determined by the individual surgeon. Generally, wedge resection was performed for peripheral nodules when a wide resection margin was achieved, whereas segmentectomy was performed for relatively central tumors. The operative tolerance of patients was determined by pulmonary function testing, blood gas analysis, echocardiography, and electrocardiogram. Lymph node (LN) status was regularly assessed by applying thoracic CT. Distant metastasis was evaluated using brain-enhanced magnetic resonance imaging (MRI), bone scintigraphy, and ultrasound. Positron emission tomography (PET)-CT was performed for the selected patients. All the patients were staged by the 8th edition of the American Joint Committee on Cancer (AJCC) Cancer Staging Manual.

### Surgical Technique

Both the RATS and VATS sub-lobar resections were performed following the previous procedure reported by our team without separating ribs.^24,25^ Patients received double-lumen intubation and selective ventilation of the lung with general anesthesia and were placed in a lateral decubitus position. The RATS procedure was performed by adopting the da Vinci Robotic Surgical System S, Si, or Xi (Intuitive Surgical, Inc., Mountain View, CA, USA) via four minimal incisions (the S version has been out of service since 2019, when it reached the end of its designated life span), and individual thoracic surgeons determined which da Vinci Surgical System to use for RATS.

First, a 12-mm camera port was made at the eighth intercostal space (ICS) along the posterior axillary line, through which a 30° stereoscopic camera was introduced. Then, two 8-mm incisions were symmetrically created at the seventh and ninth ICSs along mid-axillary and infrascapular lines, respectively. Finally, a 4-cm incision was made at the fourth or fifth ICS along the anterior axillary line for the bedside assistant to retract lung tissues and expose operative fields.

For VATS, three or four minimal incisions were conventionally used. First, the camera port was created at the sixth or seventh ICS along the anterior axillary line. Then, two ports were made at the fourth or fifth and sixth ICSs behind the pectoralis major and along the posterior axillary line, respectively. If deemed necessary, a fourth incision was created at the ninth ICS on the posterior axillary line for assistance.

For wedge resection, preoperative CT-guided tumor localization with a hook wire was performed, and pulmonary nodules were resected with a surgical margin of at least 15 mm using the endoscopic stapler. For segmentectomy, the intersegmental fissures were divided, and the target segmental pulmonary artery, vein, and bronchus were dissected. Lymph nodes around the segmental artery and bronchus also could be removed. Then, the intersegmental plane was stapled by the endoscopic stapler.

Conventionally, during RATS sub-lobar resection, a 45-mm manual or electric stapler (Ethicon Surgical Technologies, J & J MedTech, Johnson & Johnson Co. Ltd, Bridgewater, NJ, USA), identical to that used in VATS procedures, was used with the assistance of a bedside assistant, and the robotic stapler was not used. The intraoperative rapid frozen section was performed for all patients to confirm the sufficient resection margin. The chest cavity was closed with one 24-Fr chest tube left after confirmation of no active bleeding or air leak. Conversion was defined as the operation starting with RATS or VATS dissection and finishing as rib-spreading thoracotomy.

### Outcome Evaluation and Follow-up Assessment

The 30-day postoperative complications were categorized according to the Clavien-Dindo classification system as follows: grade I (any deviation from the ordinary postoperative course without the necessity for pharmacologic or operational intervention, or merely a need for drugs such as analgesics, antipyretics, antiemetics, diuretics, or electrolytes), grade II (complication requiring pharmacologic treatment, including blood transfusion and total parenteral nutrition), grade III (comorbidities requiring surgical or endoscopic intervention), grade IV (severe complication requiring intensive care unit [ICU] treatment), and grade V (patient death).^26^

The lifetime patient follow-up assessment was designed for all patients with varied strategies per the following plan: 1 month after the surgery, every half year for the next 5 years, and annually afterward. Telephone follow-up assessment was performed yearly until death or July 2023 for patients who did not regularly visit the outpatient clinic. The latest electronic medical profiles were recorded if patients were lost to follow-up evaluation. Overall survival (OS) was calculated from operation to death and recurrence-free survival (RFS) from operation to any recurrence. Patients who died of non-NSCLC-related factors were deemed event-free when RFS was calculated.

### Statistical Analysis

Continuous variables were expressed using median and interquartile range (IQR) or mean ± standard deviation (SD), whereas categorical variables were defined using frequencies and percentages. The Kolmogorov–Smirnov test was performed to determine the distribution and homogeneous variance of continuous variables. For variables with a normal distribution and homogeneous variance, Student’s *t* test was used for comparisons. Otherwise, the Mann–Whitney *U* test was used. Categorical variables were compared using Pearson’s chi-square or Fisher’s exact test. Survival data were analyzed using Kaplan-Meier curves and the log-rank (Mantel–Cox) test. A two-sided *P* value lower than 0.05 was considered statistically significant.

Propensity score-matching (PSM) with a caliper of 0.02 was performed to establish a RATS versus VATS ratio of 1:4 based on pivotal baseline characteristics including gender, age, body mass index (BMI), comorbidities, smoking history, percentage of predicted forced expiratory volume in 1 s (FEV1), percentage of predicted diffusing capacity for carbon monoxide (DLCO), percentage of predicted vital capacity (VC), tumor location, radiographic tumor type, tumor size, tumor histology, and pathologic T, N, and tumor-node-metastasis (TNM) stages. Statistical analyses and PSM were performed with IBM SPSS Statistics v.26.0 (IBM Corporation, Armonk, NY, USA). GraphPad Prism 9 (GraphPad Software Inc., San Diego, CA, USA) was used to analyze survival data.

## Results

### Baseline Clinicopathologic Characteristics

As expressed in Table 1, the study identified of 594 eligible patients. Of these patients, 48 underwent RATS and 546 underwent VATS. Generally, the patients in the RATS and VATS groups were associated with comparable baseline clinicopathologic characteristics (all *P* > 0.050). To mitigate potential selection bias in case selection, PSM then was applied with a RATS versus VATS ratio of 1:4, leading to 45 patients in the RATS group and 180 patients in the VATS groups. Consequently, all the patient baseline characteristics were well-balanced after PSM (all *P* > 0.050).

**Table 1:** Patient baseline characteristics before and after propensity score-matching (PSM)^a^

Characteristic	Unmatched cohort			Matched cohort-		
	RATS (*n* = 48) *n* (%)	VATS (*n* = 546) *n* (%)	*P* Value	RATS (*n* = 45) *n* (%)	VATS (*n* = 180) *n* (%)	*P* value
Gender						
Male Female	28 (58.33) 20 (41.67)	312 (57.14) 234 (42.86)	0.873	26 (57.78) 19 (42.22)	102 (56.67) 78 (43.33)	0.893
Mean age (years)	81.90 ± 1.88	81.63 ± 1.67	0.361	81.93 ± 1.92	81.92 ± 1.90	0.925
Mean BMI (kg/m^2^)	23.43 ± 3.10	23.25 ± 2.89	0.838	23.37 ± 3.11	23.15 ± 2.86	0.856
Smoking history						
Ever Never	19 (39.58) 29 (60.42)	209 (38.28) 337 (61.72)	0.859	18 (0.40) 27 (0.60)	71 (39.44) 109 (60.56)	0.946
Mean pulmonary function, mean						
FEV1 (% of predicted) DLCO (% of predicted) VC (% of predicted)	90.02 ± 23.22 92.06 ± 17.82 91.50 ± 18.36	89.51 ± 17.79 90.03 ± 19.71 88.81 ± 17.46	0.450 0.558 0.255	89.66 ± 23.21 91.46 ± 17.82 90.82 ± 18.36	89.70 ± 17.32 91.16 ± 19.98 89.32 ± 18.56	0.495 0.773
Comorbidities						
None Single Multiple (≥2)	28 (58.33) 13 (27.08) 7 (14.58)	308 (56.41) 140 (25.64) 98 (17.95)	0.841	26 (57.78) 13 (28.89) 6 (13.33)	101 (56.11) 53 (29.44) 26 (14.44)	0.974
Resection approach						
Segmentectomy Wedge resection	34 (70.83) 14 (29.17)	314 (57.51) 232 (42.49)	0.072	31 (68.89) 14 (31.11)	121 (67.22) 59 (32.78)	0.831
Tumor location						
Left upper lobe Left lower lobe Right upper lobe Right middle lobe Right lower lobe	7 (14.58) 5 (10.42) 27 (56.25) 2 (4.17) 7 (14.58)	160 (29.30) 59 (10.81) 241 (44.14) 23 (4.21) 63 (11.54)	0.208	7 (15.56) 5 (11.11) 25 (55.56) 1 (2.22) 7 (15.56)	31 (17.22) 20 (11.11) 99 (55.00) 3 (1.67) 27 (15.00)	1.000
Radiographic tumor type						
Pure solid Mixed, solid dominant Mixed, GGO dominant	23 (47.92) 9 (18.75) 16 (33.33)	287 (52.56) 88 (16.12) 171 (31.32)	0.808	22 (48.89) 8 (17.78) 15 (33.33)	91 (50.56) 31 (17.22) 58 (32.22)	0.980
Tumor histology						
MIA IAC SCC/others	10 (20.83) 33 (68.75) 5 (10.42)	115 (21.06) 380 (69.60) 51 (9.34)	0.914	9 (20.00) 32 (71.11) 4 (8.89)	35 (19.44) 130 (72.22) 15 (8.33)	1.000
Mean tumor size (mm)	16.23 ± 5.03	17.73 ± 6.22	0.129	16.11 ± 5.03	16.28 ± 6.17	0.895
Pathologic T stage						
1a(mi) 1a 1b 1c 2a	10 (20.83) 5 (10.42) 26 (54.17) 5 (10.42) 2 (4.17)	115 (21.06) 45 (8.24) 302 (55.31) 70 (12.82) 14 (2.56)	0.848	9 (20.00) 5 (11.11) 24 (53.33) 5 (11.11) 2 (4.44)	35 (19.44) 17 (9.44) 100 (55.56) 21 (11.67) 7 (3.89)	0.993
Pathologic N stage						
0 1 2	46 (95.83) 1 (2.08) 1 (2.08)	532 (97.44) 8 (1.47) 6 (1.10)	0.376	44 (97.78) 1 (2.22) 0	176 (97.78) 4 (2.22) 0	1.000
Pathologic TNM stage						
IA1 IA2 IA3 IB IIA IIB IIIA	15 (31.25) 25 (52.08) 4 (8.33) 2 (4.17) 0 1 (2.08) 1 (2.08)	158 (28.94) 294 (53.85) 67 (12.27) 13 (2.38) 0 8 (1.47) 6 (1.10)	0.608	14 (31.11) 24 (53.33) 4 (8.89) 2 (4.44) 0 1 (2.22) 0	55 (30.56) 99 (55.00) 15 (8.33) 7 (3.89) 0 4 (2.22) 0	1.000

RATS, robot-assisted thoracoscopic surgery; VATS, video-assisted thoracoscopic surgery; BMI, body mass index; FEV1, forced expiratory volume in 1 s; DLCO, diffusing capacity for carbon monoxide; VC, vital capacity; GGO, ground-glass opacity; MIA, minimally invasive adenocarcinoma; IAC, invasive adenocarcinoma; SCC, squamous cell carcinoma

^a^Categorical data are expressed as *n* (%), and continuous data as mean ± standard deviation.

### Perioperative Outcomes

As shown in Table 2, RATS led to less intraoperative blood loss (60 mL [IQR, 50–100 mL] vs. 80 mL [IQR, 50–100 mL]; *P* = 0.027) and a shorter postoperative hospital stay (4 days [IQR, 3–5 days] vs. 5 days [IQR, 4–6 days]; *P* = 0.041) than VATS. Additionally, the two groups were comparable concerning the surgical duration (83.61 ± 28.17 min vs. 80.17 ± 29.50 min; *P* = 0.294), conversion rate (2.22% vs. 1.67%; *P* = 1.000), blood transfusion (0.00% vs. 1.67%; *P* = 1.000), and chest tube volume (475 mL [IQR, 340–665 mL] vs. 510 mL [IQR, 375–785 mL]; *P* = 0.387) and duration (3 days [IQR, 3–5 days] vs. 4 days [IQR, 3–5 days]; *P* = 0.368). The RATS and VATS approaches also had similar LN dissection (*P* = 0.062). Finally, the patients in the RATS and VATS groups were associated with a comparable incidence of overall (20.00% vs. 26.11%; *P* = 0.396) or any individual (all *P* > 0.050) postoperative complications.

**Table 2:** Perioperative outcomes of the matched cohort^a^

Characteristic	RATS (*n* = 45) *n* (%)	VATS (*n* = 180) *n* (%)	*P* Value
Mean surgical duration (min)	83.61 ± 28.17	80.17 ± 29.50	0.294
Conversion to thoracotomy	1 (2.22)	3 (1.67)	1.000
Median blood loss: mL (IQR)	60 (50–100)	80 (50–100)	0.027
Blood transfusion	0	3 (1.67)	1.000
Median chest tube drainage			
Volume: mL (IQR) Duration: days (IQR)	475 (340–665) 3 (3–5)	510 (375–785) 4 (3–5)	0.387 0.368
Median postoperative hospital stay: days (IQR)	4 (3–5)	5 (4–6)	0.041
30-Day postoperative complications Clavien-Dindo grades I–II Atrial fibrillation Air leak Pneumonia Pleural effusion Clavien-Dindo grades III–IV Air leak Pneumonia Pleural effusion Clavien-Dindo grade V	9 (20.00) 8 (17.78) 3 (6.67) 3 (6.67) 1 (2.22) 1 (2.22) 1 (2.22) 0 0 1 (2.22) 0	47 (26.11) 38 (21.11) 10 (5.56) 12 (6.67) 6 (3.33) 10 (5.56) 8 (4.44) 2 (1.11) 2 (1.11) 4 (2.22) 1 (0.56)	0.396 0.620 0.727 1.000 1.000 0.698 0.691 1.000 1.000 1.000 1.000
LN dissection			
LND0 LND1 LND2	5 (11.11) 12 (26.67) 28 (62.22)	50 (27.78) 35 (19.44) 95 (52.78)	0.062

RATS, robot-assisted thoracoscopic surgery; VATS, video-assisted thoracoscopic surgery; IQR, interquartile range; LN, lymph node; LND0, no LN dissection; LND1, merely hilar LN dissection; LN2: mediastinal LN dissection

^a^Continuous data are shown as mean ± standard deviation or median [IQR], and categorical data as number (%).

Furthermore, the study compared RATS and VATS within patient subgroups that underwent either segmentectomy (Table S1) or wedge resection (Table S2). In the segmentectomy subgroup, RATS reduced blood loss (60 mL [IQR, 50–100 mL] vs. 80 mL [IQR, 50–100 mL]; *P* = 0.004) and shortened the postoperative hospital stay (4 days [IQR, 3–5 days] vs. 5 days [IQR, 4–6 days]; *P* = 0.027) compared with VATS.

Additionally, RATS exhibited a tendency toward fewer postoperative complications, although the difference was not statistically significant (22.58% vs. 32.23%; *P* = 0.297). In the wedge resection subgroup, RATS significantly increased the rate of LN assessment, particularly for mediastinal LNs (57.14% vs. 16.95%; *P* = 0.008) compared with VATS. Nevertheless, RATS tended to have a longer surgical time than VATS (75.75 ± 22.24 min vs. 66.69 ± 14.93 min; *P* = 0.182).

### Long-Term Survival

**Fig. 2:**
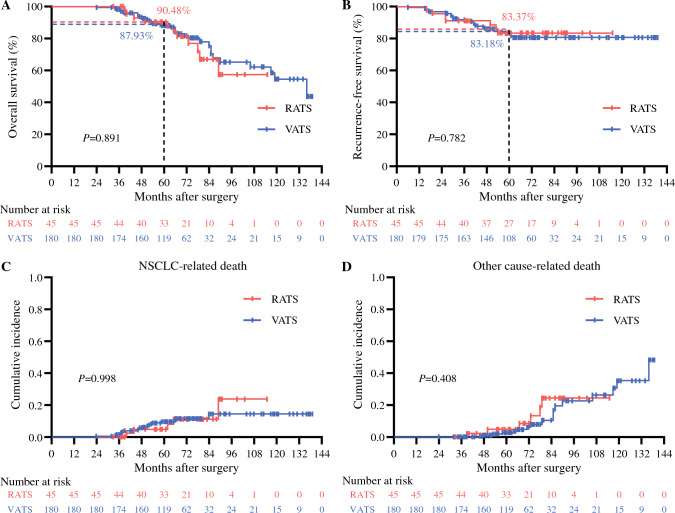
Long-term outcomes for octogenarians with early-stage small NSCLC receiving sub-lobar resection. Kaplan-Meier analysis of (**A**) OS and (**B**) RFS between the RATS and VATS groups. Analysis of cumulative incidences of (**C**) NSCLC-related death and (**D**) death related to other causes between the RATS and VATS groups. NSCLC, non-small cell lung cancer; OS, overall survival; RFS, recurrence-free survival; RATS, robot-assisted thoracoscopic surgery; VATS, video-assisted thoracoscopic surgery

As shown in Fig. 2A and B, during a median survival duration of 66 months (IQR, 55–75 months), RATS and VATS achieved comparable 5-year OS (90.48% vs. 87.93%; *P* = 0.891) and RFS (83.37% vs. 83.18%; *P* = 0.782) rates. Additionally, the RATS and VATS groups were associated with comparable cumulative incidences of NSCLC-related death (*P* = 0.998; Fig. 2C) and death related to other causes (*P* = 0.408; Fig. 2D).

**Fig. 3:**
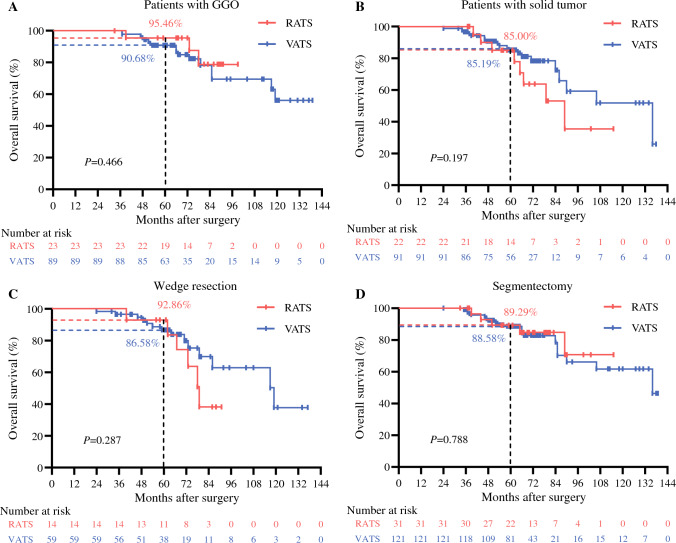
Subgroup Kaplan-Meier analysis of OS for octogenarians with early-stage small NSCLC. Comparison between RATS and VATS patients with (**A**) GGO or (**B**) solid tumor, and between RATS and VATS patients receiving (**C**) wedge resection or (**D**) segmentectomy. OS, overall survival; NSCLC, non-small cell lung cancer; RATS, robot-assisted thoracoscopic surgery; VATS, video-assisted thoracoscopic surgery; GGO, ground-glass opacity

**Fig. 4:**
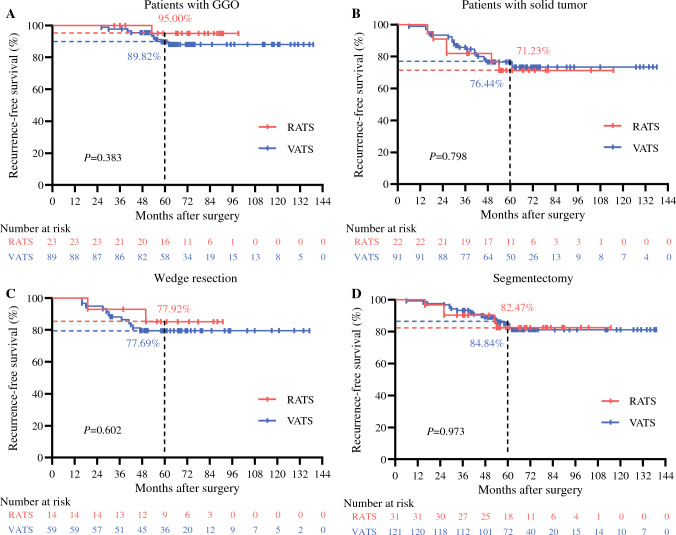
Subgroup Kaplan-Meier analysis of RFS for octogenarians with early-stage NSCLC. Comparison between RATS and VATS patients with (**A**) pure/part-solid GGO or (**B**) pure solid tumor, and between RATS and VATS patients receiving (**C**) wedge resection or (**D**) segmentectomy. RFS, recurrence-free survival; NSCLC, non-small cell lung cancer; RATS, robot-assisted thoracoscopic surgery; VATS, video-assisted thoracoscopic surgery; GGO, ground-glass opacity

Furthermore, the patients were divided into subgroups based on the radiographic tumor type (ground-glass opacity [GGO] or solid) and resection approach (segmentectomy or wedge resection). By comparison, no difference was found between RATS and VATS concerning OS (Fig. 3A–D) or RFS (Fig. 4A–D) profile within any of the subgroups (all *P* > 0.050).

Finally, multivariable Cox regression analysis showed that the surgical approach was not independently correlated with OS (*P* = 0.697) or DFS (*P* = 0.960) (Table 3). Nevertheless, the radiographic solid tumor and tumor larger than 15 mm were associated with a poorer prognosis than radiographic GGO and tumor size of 15 mm or smaller, respectively.

**TABLE 3:** Multivariable Cox regression analysis of overall survival and recurrence-free survival

Characteristic	Reference		*P* value	HR	95% CI
Overall survival					
Operative method	RATS	VATS	0.697	0.872	0.437–1.738
Gender	Female	Male	0.804	1.078	0.596–1.947
Age (years)	≥83	<83	0.607	0.840	0.433–1.632
Smoking history	Never	Ever	0.533	1.218	0.655–2.268
FEV1 (%)	≤80	>80	0.692	1.141	0.595–2.187
Tumor size (mm)	≤15	>15	0.007	2.398	1.263–4.550
Radiographic type	Solid	GGO	0.024	0.491	0.265–0.912
Procedure	Wedge resection	Segmentectomy	0.349	0.508	0.283–0.912
Recurrence-free survival					
Operative method	RATS	VATS	0.960	1.021	0.448–2.328
Gender	Female	Male	0.933	1.029	0.534–1.981
Age (years)	≥83	<83	0.823	0.920	0.445–1.905
Smoking history	Never	Ever	0.823	1.078	0.557–2.090
FEV1 (%)	≤80	>80	0.527	1.258	0.618–2.558
Tumor size (mm)	≤15	>15	0.029	1.966	1.343–3.492
Radiographic type	Solid	GGO	0.005	0.276	0.129–0.588
Procedure	Wedge resection	Segmentectomy	0.836	1.073	0.552–2.084

HR, hazard ratio; CI, confidence interval; RATS, robot-assisted thoracoscopic surgery; VATS, video-assisted thoracoscopic surgery; FEV1, forced expiratory volume in 1 s; GGO, ground-glass opacity

## Discussion

Currently, sub-lobar resection is prevalently applied with well-established feasibility and oncologic efficacy for early-stage small NSCLC.^5,6^ With the age at first diagnosis at approximately 70 years, most NSCLC patients are elderly, and many studies have confirmed that sub-lobar resection is an alternative option with improved short-term outcomes and curability comparable with lobectomy for very old patients with stage IA NSCLC.^7,8,10,27^ As an innovative MIS technique, RATS offers benefits in terms of perioperative outcomes and long-term survival similar to VATS in lobectomy and segmentectomy performed for early-stage NSCLC. However, comparisons of RATS and VATS for octogenarians with stage IA disease are scarce. This study found that RATS could reduce blood loss, decrease postoperative hospitalization, and achieve long-term outcomes comparable with VATS in sub-lobectomy performed for octogenarians with clinically early-stage NSCLC (tumor size, ≤ 2 cm).

When considering surgical-related outcomes, our study indicated that RATS reduced intraoperative blood loss, particularly in segmentectomy, compared with VATS. The superiority of RATS over VATS in reducing intraoperative blood loss might be attributable to the advantages offered by the robot-assisted surgical system, including a 3D, high-definitional surgical view and robotic arms with exceptional maneuverability, dexterity, and stability.^3,21,28^ These features mitigate the natural hand tremors, enabling surgeons to perform more precise anatomic dissections of blood vessels and bronchi, thereby avoiding unnecessary damage and excessive bleeding. Nevertheless, bleeding control is a significant benefit of sub-lobectomy over lobectomy, and patients undergoing MIS sub-lobar resection are commonly associated with minimal blood loss.^15,24,29^

Additionally, in our study, VATS had a conversion rate similar to RATS, and both achieved excellent bleeding control with minimal intraoperative blood loss, low incidences of blood transfusion, and few bleeding-relevant postoperative complications. For these reasons, both surgical procedures are safe and effective concerning bleeding control, and the reduced blood loss during RATS is clinically insignificant. Given these characteristics, intraoperative bleeding management may not be a notable clinical disadvantage of VATS versus RATS in sub-lobar resection performed for octogenarians, although VATS was associated with increased blood loss. Moreover, our findings suggested a trend toward longer surgical time with RATS than with VATS for wedge resection, despite a similar duration of segmentectomy with both procedures, which may be attributable to the benefits of RATS in anatomic dissection but the trade-off of a prolonged docking time.

Moreover, the study results showed that RATS led to a shorter postoperative hospital stay than VATS, which could be attributable to the excellent flexibility of robotic arms and high-quality surgical view that enables a more precise resection, thus minimizing unnecessary damage to normal tissues. This conjecture is evidenced by the significantly reduced blood loss and slightly decreased surgery-related complications of RATS versus VATS.

Many previous publications have compared postoperative recovery between RATS and VATS in sub-lobar resection performed for early-stage NSCLC but have drawn distinct conclusions. Zhou et al.^24^ showed that RATS could reduce postoperative ICU and hospital stays. However, the other four studies failed to observe this benefit of RATS.^30–33^ Because none of these studies focused on elderly patients, further analysis enrolling more participants 80 years old or older is necessary to validate our results.

Additionally, the study findings indicated that RATS wedge resection significantly increased the rate of LN assessment, particularly for mediastinal LNs compared with VATS. However, less than 20% of the patients who underwent VATS wedge resection received mediastinal LN dissection.

In our real-world clinical practice, systematic mediastinal LN dissection was not mandatory for sub-lobar resections. Instead, the decision regarding LN assessment was made by the individual surgeon during the procedure. Given the heightened surgical risk and prolonged postoperative recovery associated with elderly patients compared with younger individuals, there was a tendency to opt for a more conservative approach to LN dissection after comprehensive preoperative evaluation of those older than 80 years. Systematic or selective mediastinal LN assessment was typically performed for patients with pure solid or solid-dominant nodules. However, to simplify surgical procedures for wedge resection, it may be omitted for patients with the selected nodules characterized by a substantially low risk of mediastinal LN metastasis such as a consolidation-to-tumor ratio lower than 0.5, a diameter smaller than 1.0 cm, a peripheral location, and negative hilar LNs indicated by intraoperative frozen section. This strategy was particularly relevant for patients older than 80 years who were typically at higher risk during anesthesia and surgery, and has been considered sufficient in terms of oncologic efficacy for small GGO-dominant nodules.^8,15,34–36^ Meanwhile, preservation of non-involved LNs has the potential to facilitate immune activation against micro-metastatic tumor lesions and also may enhance the effectiveness of immunotherapy after recurrence.^37,38^

Additionally, although VATS generally is effective in LN dissection, the inconvenience associated with LN assessment in certain cases during wedge resection could deter thoracic surgeons from performing mediastinal LN sampling when it is anticipated to lead to heightened bleeding and extended surgical duration. This may further explain why a low proportion of patients undergoing VATS wedge resection received mediastinal dissection. Given this drawback, a more convenient process is likely to improve the willingness of thoracic surgeons to harvest more LNs, even if this is not mandatory. The da Vinci Surgical System is among the most advanced, complex, and costly pieces of surgical equipment globally, offering several inherent advantages, including a 3D, high-definition, and 10-fold magnified surgical view as well as robotic arms with full rotational capability within the chest cavity.^3,20,39^ These features provide exceptional maneuverability and enhanced dexterity compared with traditional two-dimensional VATS techniques, providing surgeons with enhanced convenience when dissecting LNs, particularly mediastinal LNs, which may be challenging to assess using VATS due to its limited flexibility. This may clarify the rise in the proportion of patients undergoing RATS wedge resection who underwent the mediastinal LN assessment. Indeed, the surgeon’s experience also could influence the LN dissection during the operation. Thus, further research to mitigate the impact of differing operative experiences among various thoracic surgeons may be required to substantiate our findings.

In terms of long-term outcomes, our results showed that RATS and VATS sub-lobectomy achieved comparable 5-year OS and RFS, consistent with two previous studies despite enrollment of younger patients. Thus, the two surgical approaches possess equal oncologic efficacies for octogenarians with early-stage NSCLC.^24,40^ Moreover, the 5-year OS and RFS of the patients in our study were approximately 89% and 83%, respectively. According to previously published studies, among very old patients with stage IA NSCLC who received sub-lobar resection, the 5-year OS and RFS ranged respectively from about 60% to 93% and about 60% to 82%.^8,10,14,27,41^ The distinct prognosis of patients indicated in various studies might be attributable to the various consolidation tumor ratios and the uneven medical conditions among different regions.

Finally, most elderly patients with early-stage NSCLC expect a postoperative survival duration of more than 5 years, so their emotional state and quality of life are essential. The RATS approach has been found to reduce long-term surgery-related pain and lead to a higher life quality compared with VATS for treating NSCLC.^42,43^ However, the long-term postoperative life-quality data are unavailable in the current research, and therefore, further investigation is needed to assess whether this RATS benefit exists for our participants.

Recently, JCOG1211, a multi-center, single-arm, confirmatory, phase 3 trial enrolling 396 cases, has shown a 5-year RFS of 98% among patients with predominantly GGO NSCLC with a tumor size of 3 cm or smaller who underwent segmentectomy.^29^ Another multi-center prospective study showed that sub-lobectomy achieved 5-year survival profiles comparable with lobectomy for octogenarians with a NSCLC tumor size of 2–4 cm.^15^ These promising results suggest that sub-lobar resection is a promising method for patients with NSCLC tumors larger than 2 cm. Given these findings, further determining the safety, feasibility, and oncologic efficacy RATS sub-lobar resection for very old patients who have early-stage NSCLC with a tumor diameter larger than 2 cm might expand its application in treating NSCLC.

Some limitations of this study are acknowledged. First, the representative nature of the study could have led to potential bias in patient selection, although PSM was performed and the patient baseline characteristics were well-balanced. Therefore, further prospective study is necessary to validate our findings.

Second, this study represented the real-world practice of a single center that included only Chinese patients. Given this exclusive study population, the conclusion obtained universally requires further confirmation by an international study with a large patient sample.

Third, the diverse surgical experiences, the varying levels of surgical expertise, and the learning curve associated with RATS and VATS among different thoracic surgeons at our medical center may have influenced perioperative and even long-term outcomes. Nevertheless, managing these variables in a retrospective study is challenging. Hence, analyzing the impact of surgeon-related factors on patient outcomes and comparing RATS and VATS performed by one experienced and highly skilled thoracic surgeon may require additional research.

Finally, the postoperative pulmonary function data are not available in our center. Thus, the efficacies of pulmonary function preservation between RATS and VATS need further investigation.

## Conclusions

In summary, RATS could shorten the postoperative hospital stay, reduce blood loss by a statistically notable but clinically insignificant amount, and achieve long-term outcomes comparable with VATS in performing sub-lobar resection for octogenarians with small early-stage NSCLC.

### Supplementary Information

Below is the link to the electronic supplementary material.

Supplementary file 1 (DOCX 26 KB)

## Data Availability

The database analyzed during this study is available from the corresponding authors Jia Huang and Qingquan Luo upon reasonable request.
