# Supplementary material for: Robotic Versus Thoracoscopic Sub-lobar Resection for Octogenarians with Clinical Stage IA Non-small Cell Lung Cancer: A Propensity Score-Matched Real-World Study
Source: Ann Surg Oncol. 2023 Dec 10;31(3):1568–80. doi: 10.1245/s10434-023-14689-5 (PMC10838251; doi:10.1245/s10434-023-14689-5)
Supplement: Supplementary file 1 — (DOCX 26 KB) [file 10434_2023_14689_MOESM1_ESM.docx]

**Supplementary Information**

**Robotic Versus Thoracoscopic Sub-Lobar Resection for** **Octogenarians with Clinical IA-Stage Non-Small Cell Lung Cancer: A Propensity-Score Matched Real-World Study**

**Hanbo Pan, PhD^1^, Ningyuan Zou, MD^1^, Yu Tian, PhD^1^, Yaofeng Shen, MD^2^, Hang Chen, MD^3^, Hongda Zhu, MD^1^, Jiaqi Zhang, MD^1^, Weiqiu Jin, MD^1^, Zenan Gu, PhD^1^, Junwei Ning, MD^1,4^, Long Jiang, MD, PhD^1^, Jia Huang, MD, PhD^1^, and Qingquan Luo, MD, FACS, FRCS^1^**

^1^Shanghai Lung Cancer Center, Shanghai Chest Hospital, Shanghai Jiao Tong University School of Medicine, Shanghai, China

^2^Department of Anesthesiology, Shanghai Chest Hospital, Shanghai Jiao Tong University, Shanghai, China

^3^Department of Cardiothoracic Surgery, The Affiliated Lihuili Hospital of Ningbo University, Zhejiang, China

^4^Department of Thoracic Surgery, Shanghai Tongren Hospital, Shanghai Jiao Tong University School of Medicine, Shanghai, China

**TABLE S1** Perioperative outcomes of patients undergoing segmentectomy after PSM

| Characteristic | RATS (*n*=31) | VATS (*n*=121) | *P* value |
| --- | --- | --- | --- |
| Surgical duration (mins), mean ± SD | 87.16±30.12 | 86.74±32.52 | 0.734 |
| Conversion to thoracotomy, n (%) | 1 (3.23) | 3 (2.48) | 1.000 |
| Blood loss (mL), median [IQR] | 60 [50-100] | 80 [50-100] | 0.004 |
| Blood transfusion, n (%) | 0 | 3 (2.48) | 1.000 |
| Chest tube drainage, median [IQR]  Volume (mL)  Length (days) | 545 [325-659]  3 [3-5] | 580 [410-790]  4 [3-5] | 0.143  0.520 |
| Postoperative hospital stays (days), median [IQR] | 4 [3-5] | 5 [4-6] | 0.027 |
| 30-Day postoperative complications, n (%)  Clavien-Dindo Grade I-II  Atrial fibrillation  Air leak  Pneumonia  Pleural effusion  Clavien-Dindo Grade III-IV  Air leak  Pneumonia  Pleural effusion  Clavien-Dindo Grade V | 7 (22.58)  6 (19.35)  2 (6.45)  2 (6.45)  1 (3.23)  1 (3.23)  1 (3.23)  0  0  1 (3.23)  0 | 39 (32.23)  33 (27.27)  8 (6.61)  11 (9.09)  5 (4.13)  9 (7.44)  6 (4.96)  1 (0.83)  1 (0.83)  4 (3.31)  0 | 0.297  0.368  1.000  1.000  1.000  0.688  1.000  1.000  1.000  1.000  - |
| LN dissection ^a^, n (%)  LND0  LND1  LND2 | 0  11 (35.48)  20 (64.52) | 5 (4.13)  31 (25.62)  85 (70.25) | 0.385 |

Continuous data are shown as mean ± SD or median [IQR], and categorical data are expressed as number (percentage). LN dissection ^a^: LND0, no LN dissection; LND1, merely hilar LN dissection; LN2: mediastinal LN dissection. *RATS, robotic-assisted thoracoscopic surgery; VATS, video-assisted thoracoscopic surgery; SD, standard deviation; LN, lymph node; IQR,* *interquartile range**.*

**TABLE S2** Perioperative outcomes of patients undergoing wedge resection after PSM

| Characteristic | RATS (*n*=14) | VATS (*n*=59) | *P* value |
| --- | --- | --- | --- |
| Surgical duration (mins), mean ± SD | 75.75±22.24 | 66.69±14.93 | 0.182 |
| Conversion to thoracotomy, n (%) | 0 | 0 | - |
| Blood loss (mL), median [IQR] | 50 [50-90] | 50 [50-80] | 0.828 |
| Blood transfusion, n (%) | 0 | 0 | - |
| Chest tube drainage, median [IQR]  Volume (mL)  Length (days) | 445 [365-813]  3 [3-5] | 390 [310-685]  4 [3-5] | 0.492  0.634 |
| Postoperative hospital stays (days), median [IQR] | 3 [3-5] | 4 [3-5] | 0.576 |
| 30-Day postoperative complications, n (%)  Clavien-Dindo Grade I-II  Atrial fibrillation  Air leak  Pneumonia  Pleural effusion  Clavien-Dindo Grade III-IV  Air leak  Pneumonia  Pleural effusion  Clavien-Dindo Grade V | 2 (14.29)  2 (14.29)  1 (7.14)  1 (7.14)  0  0  0  0  0  0  0 | 8 (13.56)  5 (8.47)  2 (3.39)  1 (1.69)  1 (1.69)  1 (1.69)  2 (3.39)  1 (1.69)  1 (1.69)  0  1 (1.69) | 1.000  0.613  0.477  0.349  1.000  1.000  1.000  1.000  1.000  -  1.000 |
| LN dissection ^a^, n (%)  LND0  LND1  LND2 | 5 (35.71)  1 (7.14)  8 (57.14) | 45 (76.27)  4 (6.78)  10 (16.95) | 0.008 |

Continuous data are shown as mean ± SD or median [IQR], and categorical data are expressed as number (percentage). LN dissection ^a^: LND0, no LN dissection; LND1, merely hilar LN dissection; LN2: mediastinal LN dissection. *RATS, robotic-assisted thoracoscopic surgery; VATS, video-assisted thoracoscopic surgery; SD, standard deviation; LN, lymph node; IQR, interquartile range.*
